# Release of Phosphorylated HSP27 (HSPB1) from Platelets Is Accompanied with the Acceleration of Aggregation in Diabetic Patients

**DOI:** 10.1371/journal.pone.0128977

**Published:** 2015-06-05

**Authors:** Haruhiko Tokuda, Gen Kuroyanagi, Masanori Tsujimoto, Yukiko Enomoto, Rie Matsushima-Nishiwaki, Takashi Onuma, Akiko Kojima, Tomoaki Doi, Kumiko Tanabe, Shigeru Akamatsu, Hiroki Iida, Shinji Ogura, Takanobu Otsuka, Toru Iwama, Takahisa Tanikawa, Kei Ishikawa, Kumi Kojima, Osamu Kozawa

**Affiliations:** 1 Department of Clinical Laboratory, National Center for Geriatrics and Gerontology, Obu, Aichi, Japan; 2 Department of Pharmacology, Gifu University Graduate School of Medicine, Gifu, Japan; 3 Department of Orthopedic Surgery, Nagoya City University Graduate School of Medical Sciences, Nagoya, Japan; 4 Department of Neurosurgery, Gifu University Graduate School of Medicine, Gifu, Japan; 5 Department of Anesthesiology and Pain Medicine, Gifu University Graduate School of Medicine, Gifu, Japan; 6 Department of Anesthesiology and Critical Care Medicine, Matsunami General Hospital, Gifu, Japan; 7 Department of Emergency and Disaster Medicine, Gifu University Graduate School of Medicine, Gifu, Japan; Center for Cancer Research, National Cancer Institute, UNITED STATES

## Abstract

We investigated the relationship between HSP27 phosphorylation and collagen-stimulated activation of platelets in patients with diabetes mellitus (DM). Platelet-rich plasma was prepared from blood of type 2 DM patients. The platelet aggregation was analyzed in size of aggregates by an aggregometer using a laser scattering method. The protein phosphorylation was analyzed by Western blotting. Phosphorylated-HSP27 and PDGF-AB released from platelets were measured by ELISA. The phosphorylated-HSP27 levels at Ser-78 and Ser-82 induced by collagen were directly proportional to the platelet aggregation. Total HSP27 levels in platelets were decreased concomitantly with the phosphorylation. The released HSP27 levels were significantly correlated with the phosphorylated levels of HSP27 in the platelets stimulated by 0.3 μg/ml collagen. The low dose collagen-stimulated release of HSP27 was detected but relatively small in healthy donors. The released levels of PDGF-AB were in parallel with the levels of released HSP27. Area under the curve (AUC) of small aggregation (9-25 μm) induced by 0.3 μg/ml collagen was inversely proportional to the levels of released HSP27. AUC of large aggregation (50-70 μm) was directly proportional to the levels of released HSP27. Exogenous recombinant phosphorylated- HSP27 hardly affected the aggregation or the released levels of PDGF-AB induced by collagen. These results strongly suggest that HSP27 is released from human platelets accompanied with its phosphorylation induced by collagen, which is correlated with the acceleration of platelet aggregation in type 2 DM patients.

## Introduction

Accelerated thrombosis, which causes brain stroke and acute coronary syndrome including myocardial infarction, is a major health concern of the high mortality or the debilitation of daily life [1]. In those pathological states, platelet hyper-aggregation plays a central role, associated with vascular diseases [2]. The first step of platelet activation is triggered by initial tethering at the injured vascular sites exposing subendothelial collagen, characterized by the formation of glycoprotein (GP) Ib/IX/V and von Willebrand factor complex [1], [2]. Activated platelets rapidly secrete adenosine diphosphate (ADP) etc., release thromboxane A_2_ etc., and promote repairment of vascular injury [1]. The mitogenic mediators such as platelet-derived growth factor-AB (PDGF-AB) are secreted from activated platelets as granule contents and modifies vascular endothelial-cell function, resulting in the progression of atherosclerosis and vascular stenosis [1]. Collagen, by binding to integrin-α2β1 and GPVI, elicits the integrated signaling and upregulation of integrin activities in platelets, subsequently stimulating the granule secretion enhancement and the development of coagulant activity [3], [4], [5].

In 21th century, type 2 diabetes mellitus (DM) is considered as one of the global problems of human health [6]. Progression of atherosclerotic change with elevated spontaneous platelet aggregation are observed even in early stage of DM, so the patients are suffering from an increased risk of vascular complications such as cardiovascular diseases [7]. In order to improve the prognosis of DM patients, the adequate control of platelet aggregation based upon the pathogenesis of platelet functions in DM is essential. Regarding the acceleration of platelet aggregation, we have previously reported that a low-dose (1 μM) of ADP induces irreversible platelet microaggregation in the majority of type 2 DM patients, and that the P2Y12 receptors are involved in the hypersensitivity of platelet aggregation [8]. In addition, we have also shown that the acceleration of platelet aggregation stimulated by collagen is closely correlated with the activation of p44/p42 mitogen-activated protein (MAP) kinase and p38 MAP kinase in the type 2 DM patients [9]. However, the exact mechanism behind platelet hyper-aggregation in DM patients is not fully understood.

A variety of biological stresses such as heat induce the expression of heat shock proteins (HSPs) in both prokaryotic and eukaryotic cells [10]. HSPs are recognized to act as molecular chaperones protecting unfolded proteins from aggregation. Among them, low-molecular-weight HSPs (HSPB) including HSP27 (HSPB1) and αB-crystallin (HSPB5) possesses high homology in their amino acid sequences, α-crystallin domain, and principally functions as molecular chaperones [11], [12]. Accumulating evidence suggests that HSP27 expresses multiple pleiotropic functions such as anti-apoptosis [13], [14] and stabilizing actin and microtubules of cytoskeleton [12], [15]. HSP27 undergoes post-translational modification such as phosphorylation [10]. Human HSP27 is reportedly phosphorylated at three serine residues (Ser-15, Ser-78 and Ser-82) [10], [16]. HSP27 in resting state exists in an unphosphorylated aggregated form. Once phosphorylated, HSP27 is rapidly dissociated, resulting in forming a dissociated form (dimer or monomer), which is thought to be necessary for substrate binding and specific functions [10], [17]. It is generally known that the phosphorylation of HSP27 is catalyzed by members of the MAP kinase superfamily, such as p38 MAP kinase [10], [18]. In response to collagen, it has been reported that p38 MAP kinase is activated and regulates HSP27 phosphorylation in the process of human platelet activation [18]. We have recently reported that Rac, a low-molecular weight GTP-binding protein, regulates collagen-induced HSP27 phosphorylation via p44/p42 MAP kinase in human platelets, leading to the secretion of PDGF-AB [19]. However, the clinical relevance of HSP27 phosphorylation in human platelets has not yet been precisely clarified.

In the present study, we investigated the relationship between HSP27 phosphorylation and collagen-stimulated activation of platelets in type 2 DM patients. We herein show that HSP27 is released from human pletelets after the phosphorylation of HSP27 at Ser-78 induced by collagen, which is correlated with the acceleration of platelet aggregation in type 2 DM patients.

## Materials and Methods

### Materials

Collagen was purchased from Takeda Austria GmbH. (Wien, Austria). Anti-HSP27 antibodies and anti-phospho HSP27 (Ser-78) antibodies were purchased from Stressgen Biotechnologies (Victoria, BC, Canada). Anti-phospho HSP27 (Ser-82) antibodies were purchased from Biomol Research Laboratories (Plymouth Meeting, PA). Anti-P2Y12 antibodies were purechased from Santa Cruz Biotechnology, Inc. (Santa Cruz, CA). Recombinant phosphorylated-HSP27 (Ser-15, Ser-78, Ser-82) was obtained from Enzo Life Science, Inc. (Farmingdale, NY). The PDGF-AB enzyme-linked immunosorbent assay (ELISA) kit was purchased from R&D (Minneapolis, MN). The phosphorylated-HSP27 ELISA kit was purchased from Enzo Life Science, Inc. (Plymouth Meeting, PA). Other materials and chemicals were obtained from commercial sources.

### Subjects

The inclusion criteria for the study were the presence of type 2 DM according to the criteria of the World Health Organization. We excluded the patients who were complicated with a malignancy, infectious diseases including hepatitis B and hepatitis C, or autoimmune disorders. All participants were advised to avoid sleep deprivation or blood donation. The study was approved by the committee of the conduct of human research at National Center for Geriatrics and Gerontology and at Gifu University Graduate School of Medicine. Written informed consent was obtained from all of the patients and healthy donors.

### Blood sampling

Ten ml of blood was drawn from the vein between 8:00 and 9:00 after at least 15 min of bed rest to preserve steady state conditions. Sodium citrate (14 μM) was added to the blood immediately as an anticoagulant, and platelet-rich plasma (PRP) was obtained by centrifugation at 155 x *g* for 12 min at room temperature. Platelet-poor plasma (PPP) was prepared from the residual blood by centrifugation at 1,400 x *g* for 5 min.

### Platelet aggregation

Platelet aggregation was measured using an aggregometer (PA-200 apparatus, Kowa Co. Ltd., Tokyo, Japan) with a laser-scattering system as described previously [8], [9]. In brief, PRP was preincubated at 37°C for 1 min with a stirring speed of 800 rpm. Platelet aggregation was monitored for 4 min after the addition of various doses of collagen (0, 0.1, 0.3 and 1.0 μg/ml). When indicated, platelet aggregation was monitored for 4 min after the addition of 1.0 μg/ml of collagen or vehicle with or without 3 μg/ml of recombinant phosphorylated-HSP27. The percentage of transmittance of the isolated platelets was recorded as 0%, and that of the appropriate PPP (blank) was recorded as 100%. Platelet aggregation was then terminated by the addition of ice-cold EDTA (10 mM). The conditioned mixture was collected and centrifuged at 10,000 x *g* at 4°C for 2 min. The supernatant was collected and stored at -80°C. The pellet was washed twice with PBS and then lysed immediately by boiling in a lysis buffer containing 62.5 mM Tris-HCl, pH 6.8, 2% sodium dodecyl sulfate (SDS), 50 mM dithiothreitol and 10% glycerol for a Western blot analysis.

### Western blot analysis

A Western blot analysis was performed as described previously [20]. In brief, SDS-polyacrylamide gel electrophoresis (PAGE) was performed by the method described by Laemmli [21] in a 12.5% polyacrylamide gel. The proteins fractioned in the gels were transferred onto polyvinylidene fluoride (PVDF) membranes, and then the membranes were blocked with 5% fat-free dry milk in Tris-buffered saline with 0.1% Tween-20 (TBS-T, 20 mM Tris, pH 7.6, 137 mM NaCl, 0.1% Tween-20) for 2 h before incubation with the indicated primary antibodies. Peroxidase-labeled antibodies raised in a goat against rabbit IgG (KPL, Gaithersburg, MD, USA) were used as the secondary antibodies. The primary and secondary antibodies were diluted to their optimal concentrations with 5% fat-free dry milk in TBS-T. The peroxidase activity on the PVDF membrane was visualized with X-ray film by means of an ECL Western blotting detection system (GE Healthcare, Buckinghamshire, UK) following the manufacturer’s protocol. The bands were analyzed by densitometry using the ImageJ software program (National Institutes of Health, Bethesda, MD). The quantitative data of each samples were obtained as the counts of pixels.

### ELISA for PDGF-AB and phosphorylated-HSP27

The levels of PDGF-AB and phosphorylated-HSP27 in the supernatant of the conditioned mixture after platelet aggregation were determined using ELISA kits for PDGF-AB and phosphorylated-HSP27, respectively.

### Statistical analysis

The statistical significance of the correlation between two variables, linear regression analysis was adopted using SPSS ver. 19.0 (IBM Japan Ltd., Tokyo, Japan) as a software. A probability of less than 5% was considered to be statistically significant.

## Results

### Characterization of the subjects for Western blotting and ELISA

The clinical and biochemical characteristics of the subjects (n = 35) are presented in Table 1. Among them, 27 patients were adopted for Western blot analysis. The HbA_1c_ levels of the subjects for Western blot analysis and ELISA were 8.5 ± 2.3% and 8.4 ± 2.1%, respectively, and those were significantly higher than the upper limit of normal range (5.9%). The anthropometric indexes were within the normal limits in Japanese, and the significant changes of metabolic variables were not observed.

**Table 1:** Characteristics of the study subjects.

parameters	For Western blotting	For ELISA
Total number	27	35
Gender (F/M)	16 / 11	12/23
Age (years)	68.7 ± 7.7	70.0 ± 8.0
DM duration (years)	8.4 ± 8.7	9.6 ± 9.7
Height (cm)	158.0 ± 8.2	158.0 ± 8.2
Weight (kg)	59.7 ± 13.2	60.7 ± 12.8
BMI	23.8 ± 4.6	24.2 ± 4.4
sBP (mmHg)	116.8 ± 16.5	119.2 ± 18.8
dBP (mmHg)	67.1 ± 10.4	67.6 ± 11.1
HbA1c (%)	8.5 ± 2.3	8.4 ± 2.1
Glu (mg/dl)	159.3 ± 63.6	154.3 ± 58.1
TC (mg/dl)	207.1 ± 38.0	200.8 ± 39.4
TG (mg/dl)	129.8 ± 72.6	128.7 ± 66.7
HDL (mg/dl)	50.9 ± 12.1	50.1 ± 14.0
Plt (x104/μl)	21.7 ± 5.7	22.1 ± 5.4

F indicates female; M, male; BMI, body mass index; sBP, systolic blood pressure; dBP, diastolic blood pressure; HbA_1c_, hemoglobin A_1c_; Glu, plasma glucose; TC, total cholesterol; TG, triglyceride; HDL, high-density lipoprotein; Plt, platelet counts. The data are presented as the means ± SD.

### Platelet aggregation and the phosphorylation of HSP27 in the subjects

**Fig 1:**
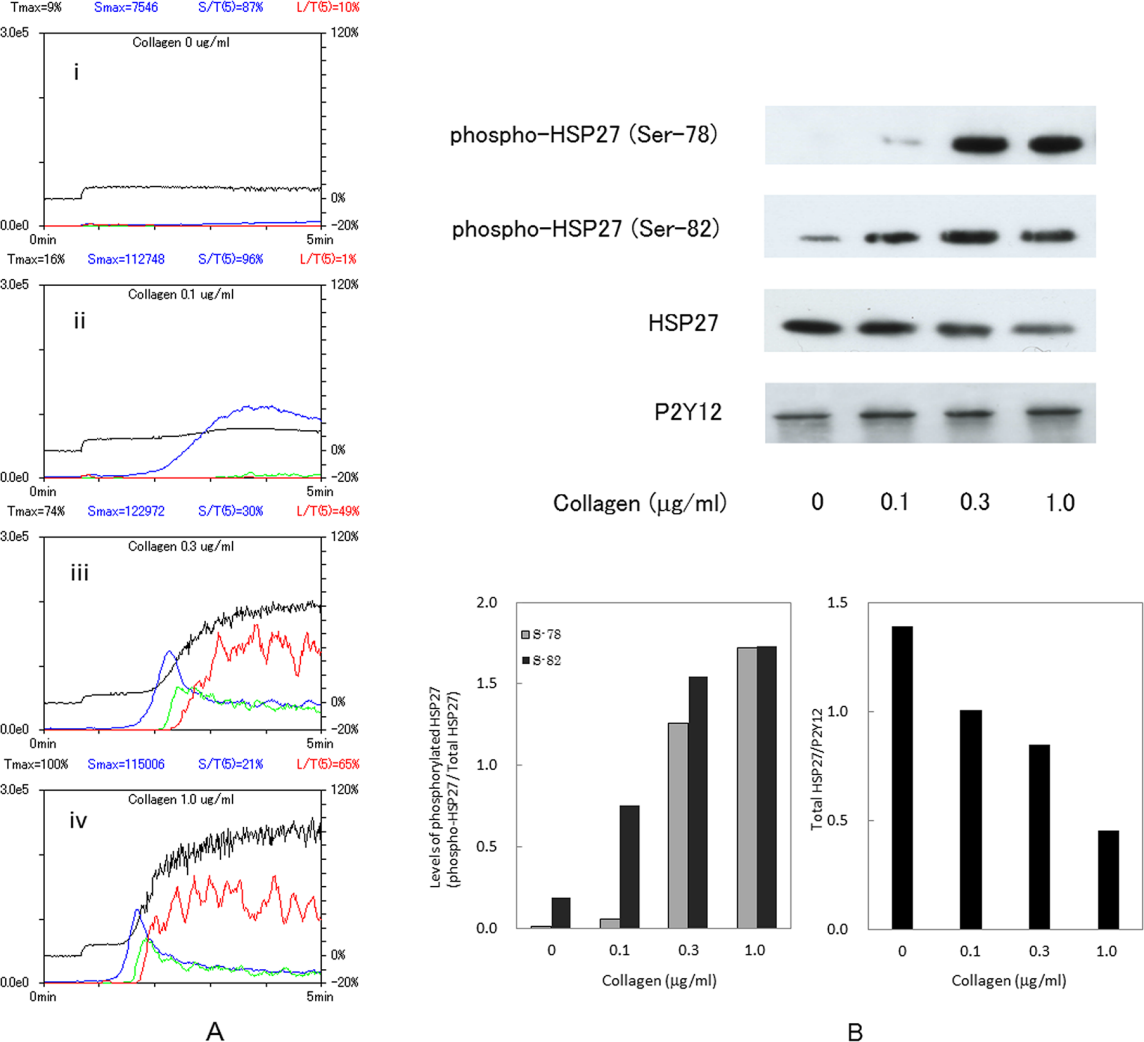
Representative patterns of platelet aggregation induced by various doses of collagen as detected by an aggregometer with laser-scattering system and representative data showing the collagen-induced HSP27 phosphorylation in platelets from type 2 DM patients. PRP from type 2 DM patients was stimulated by various doses of collagen (0, 0.1, 0.3 and 1.0 μg/ml) in an aggregometer at 37°C for 4 min with a stirring speed of 800 rpm. (A) Time-dependent changes in the platelet aggregation after stimulation by 0 μg/ml (*i*), 0.1 μg/ml (*ii*), 0.3 μg/ml (*iii*), and 1.0 μg/ml (*iv*) are shown. The black line indicates the percentage of transmittance of each samples (the isolated platelets were recorded as 0%, and platelet free plasma was recorded as 100%). The blue line indicates small aggregates (9–25 μm); green line, medium aggregates (25–50 μm); red line, large aggregates (50–70 μm). (B) The reaction was terminated by the addition of an ice-cold EDTA (10 mM) solution. The extracts of platelets were subjected to a Western blot analysis using antibodies against phospho-specific HSP27 (Ser-78 and Ser-82), total HSP27 and P2Y12. The bands were quantified using the ImageJ software program as the counts of pixels. The bands of phospho-HSP27 and the bands of total HSP27 were normalized to the total HSP27 bands and the P2Y12 bands, respectively. The ratio (phospho-HSP27/total HSP27 and total HSP27/P2Y12) is presented for each value. Upper panel indicates the results of a Western blot analysis. In the left-lower panel, the semi-black bars indicate the phosphorylation ratio of HSP27 (Ser-78), and the black bars indicate the phosphorylation ratio of HSP27 (Ser-82). In the right-lower panel, the black bars indicate the ratio of total HSP27/P2Y12.

Representative pattern of collagen-induced platelet aggregation in the study groups analyzed by an aggregometer with the laser scattering system are shown in Fig 1A. Collagen dose-dependently elicited platelet aggregation in the range between 0.1 μg/ml and 1.0 μg/ml. In the case of type 2 DM, the large platelet aggregate (50–70 μm) was significantly induced by 0.3 μg/ml collagen, and the ratio reached at 49% of total aggregates. Regarding the collagen-induced platelet aggregation in healthy subjects, we previously reported that the collagen ED_50_ value for platelet aggregation in the non-DM healthy subjects is 0.460 ± 0.082 μg/ml [9].

It is well known that human HSP27 is phosphorylated at three serine residues (Ser-15, Ser-78 and Ser-82) [10], [16]. Thus, we examined the effects of collagen on the phosphorylation of HSP27 (Ser-78 and Ser-82) by a Western blot analysis. As a result, collagen significantly induced the phosphorylations at the two serine-residues (Fig 1B). The levels of phosphorylated-HSP27 were directly proportional to the platelet aggregation. Interestingly, the total levels of HSP27 decreased in contrast to the phosphorylation (Fig 1B), suggesting that the phosphorylated-HSP27 was released from the activated platelets in response to collagen stimulation. We confirmed that the levels of P2Y12 receptor proteins, a member of GTP-binding protein-coupled receptors located on the platelet membrane [22], were not changed by the collagen stimulation in these platelets (Fig 1B).

### The relationship between individual levels of HSP27 phosphorylation and the change of intracellular HSP27 induced by collagen in the platelets of type 2 DM patients

**Fig 2:**
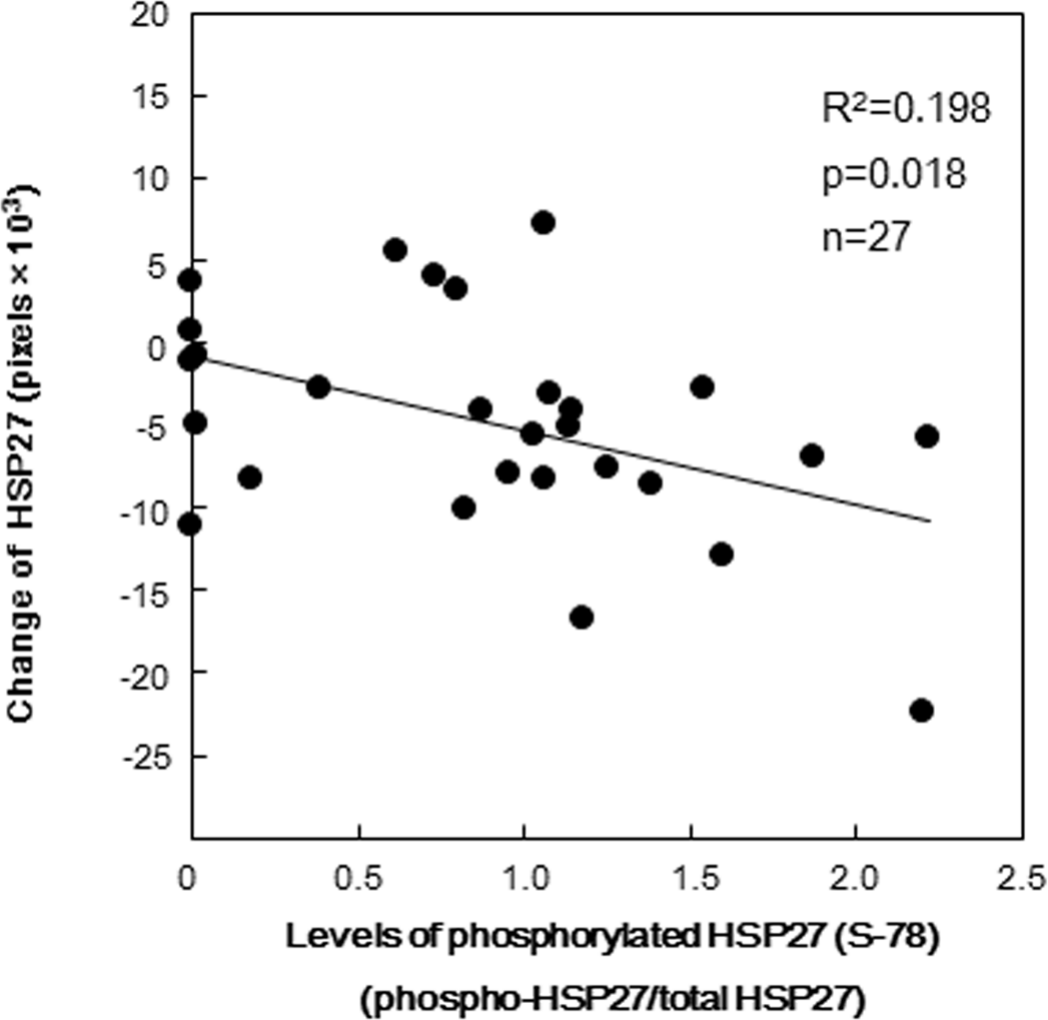
The relationship between individual levels of HSP27 phosphorylation (Ser-78) and the change of intracellular HSP27 protein levels induced by collagen in platelets from type 2 DM patients. The baseline levels in unstimulated samples were subtracted from each of the individual HSP27 phosphorylation ratio (phosphorylated-HSP27/total HSP27) and intracellular HSP27 protein levels stimulated by 0.3 μg/ml of collagen for 4 min, and the net changes are presented as levels of phosphorylated-HSP27 (Ser-78) and changes of HSP27, respectively. Each data were determined by a Western blot analysis using the ImageJ software program and were plotted and analyzed by linear regression analysis.

To investigate the ratio of phosphorylated-HSP27/total HSP27 after the collagen-stimulated platelet activation, we plotted the individual levels of HSP27 phosphorylation against the change of total HSP27 levels induced by 0.3 μg/ml collagen in the group for Western blotting. The change of total HSP27 levels in platelets was inversely correlated with the phosphorylated levels of HSP27 at Ser-78 (R^2^ = 0.198, p = 0.018) (Fig 2).

### The relationship between individual levels of HSP27 phosphorylation and released phosphorylated-HSP27 induced by collagen in the platelets of type 2 DM patients

**Fig 3:**
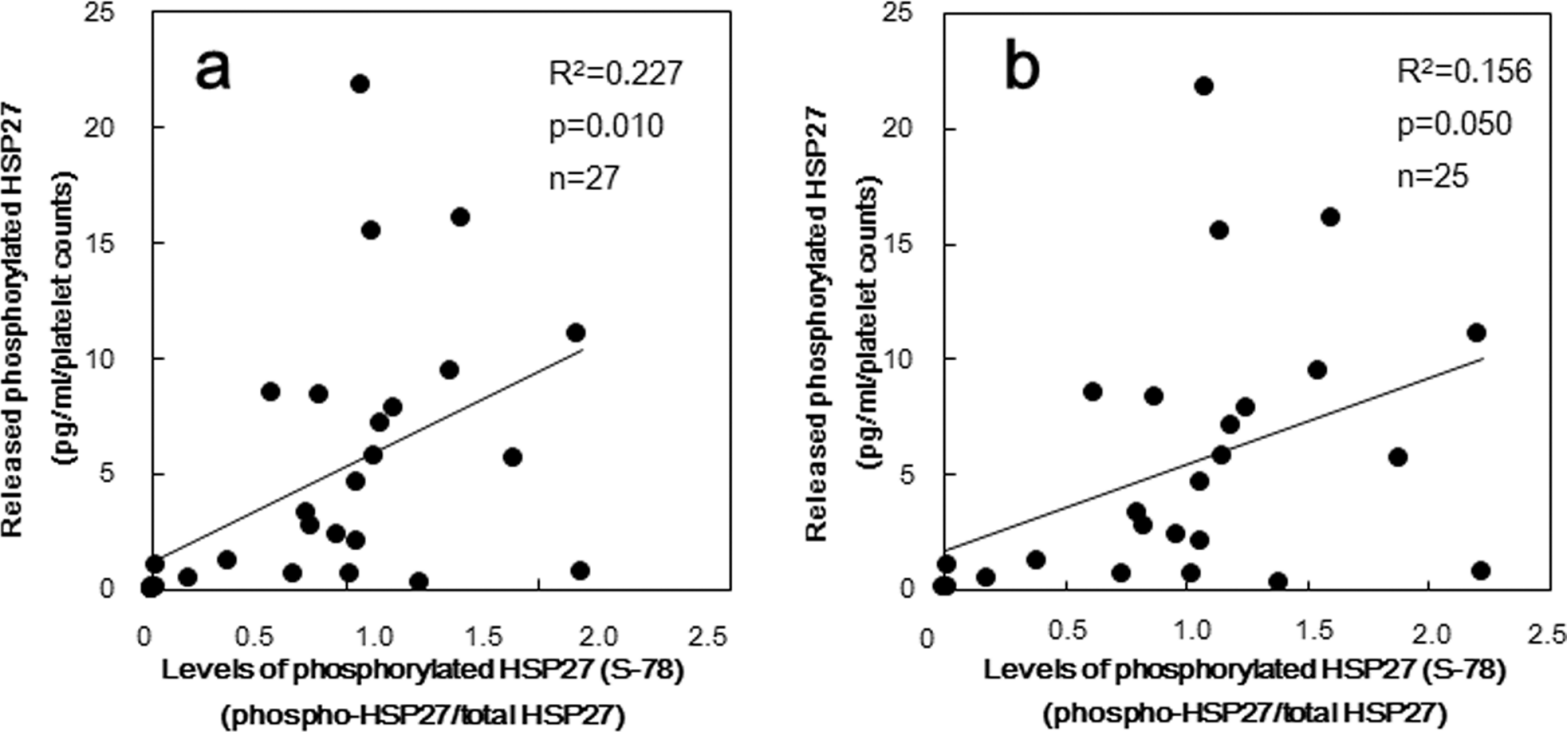
The relationship between individual levels of HSP27 phosphorylation (Ser-78) in the platelets and the released phosphorylated HSP27 levels induced by collagen in type 2 DM patients. The baseline levels in unstimulated samples were subtracted from each of the individual HSP27 phosphorylation ratio (phosphorylated-HSP27/total HSP27) and phosphorylated HSP27 levels in the conditioned mixture after platelet aggregation stimulated by 0.3 μg/ml of collagen for 30 min were collected with the platelet counts. Each data about the phosphorylation of HSP27 in platelets and the levels of released phosphorylated-HSP27 were determined by a Western blot analysis using the ImageJ software program and ELISA, respectively. These data were plotted and analyzed by linear regression analysis. (a) Whole subjects (n = 27) were plotted. (b) The residual subjects after excluding what concentration of phosphorylated-HSP27 could not be detected (n = 25) were plotted.

In order to clarify whether the inverse correlation between the change of HSP27 levels in platelets and HSP27 phosphorylation induced by collagen is due to the release of HSP27 from platelets or not, we plotted the levels of released phosphorylated-HSP27 against the individual levels of collagen-induced phosphorylation of HSP27 in the group for Western blotting. The levels of released phosphorylated-HSP27 induced by 0.3 μg/ml collagen was significantly correlated with the levels of HSP27 phosphorylation at Ser-78 (R^2^ = 0.227, p = 0.010, n = 27) (Fig 3A). As the plasma levels of phosphorylated-HSP27 were strongly affected by the counts of platelet in each sample, we presented the data adjusted by platelet counts (pg/ml/platelet counts). In the subjects of ELISA, there were 5 non-responding samples in which concentration of phosphorylated-HSP27 could not be detected. The relationship between the levels of released phosphorylated-HSP27 and the levels of HSP27 phosphorylation was slightly weaken by the exclusion of non-responding samples (R^2^ = 0.156, p = 0.050, n = 25) (Fig 3B). We also investigated the levels of released phosphorylated-HSP27 from the platelets of 5 healthy donors induced by 0.3 μg/ml collagen, and found that the levels were estimated as 1.27 ± 2.46 pg/ml/platelet counts (the baseline levels in unstimulated samples were subtracted to produce each data). The levels of low dose collagen-stimulated release of HSP27 were detectable but relatively small in non-DM subjects compared with those in type 2 DM patients.

### The relationship between individual levels of secreted PDGF-AB and released phosphorylated-HSP27 induced by collagen from the platelets of type 2 DM patients

**Fig 4:**
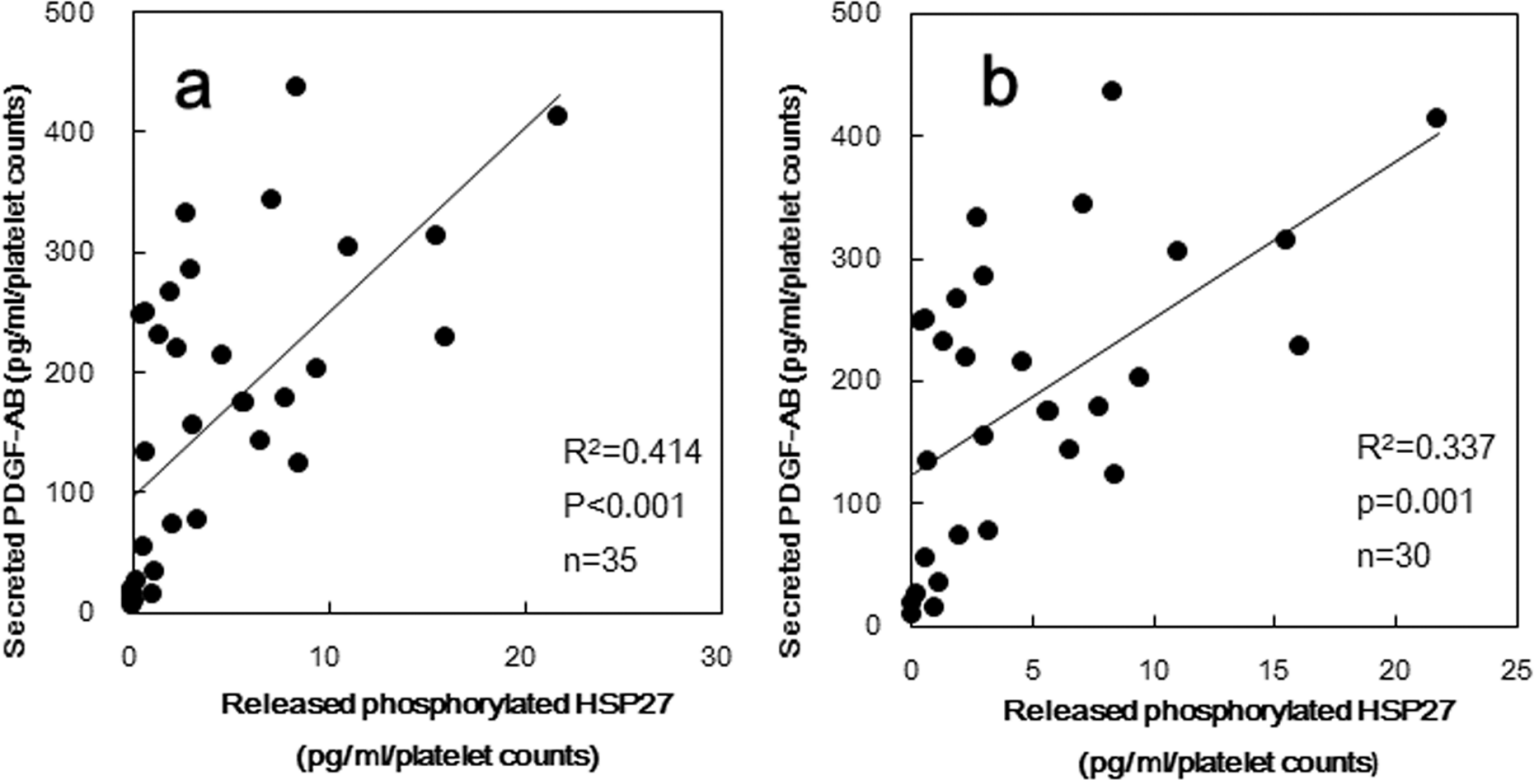
The relationship between individual levels of released phosphorylated-HSP27 and the levels of PDGF-AB induced by collagen in type 2 DM patients. The level of phosphorylated-HSP27 and PDGF-AB in the supernatant of the conditioned mixture after platelet aggregation stimulated by 0.3 μg of collagen for 30 min was determined using specific ELISA kits. The baseline levels in unstimulated samples were subtracted from each of the individual released phosphorylated-HSP27 levels and the PDGF-AB levels. Each data were collected with the platelet counts, and were plotted and analyzed by linear regression analysis. (a) Whole subjects (n = 35) were plotted. (b) The residual subjects after excluding what concentration of phosphorylated-HSP27 could not be detected (n = 30) were plotted.

PDGF-AB is recognized as a mitogenic mediator which is secreted from activated platelets and promotes the progress of atherosclerosis [1]. We have reported that collagen-induced HSP27 phosphorylation is sufficient for PDGF-AB secretion from human platelets and that the secretion is regulated by Rac [19]. Thus, we compared the collagen-induced PDGF-AB secretion against the individual levels of released phosphorylated-HSP27 from the platelets of type 2 DM patients. The levels of PDGF-AB secretion induced by 0.3 μg/ml collagen was significantly correlated with the levels of released phosphorylated-HSP27 (R^2^ = 0.414, p<0.001, n = 35) (Fig 4A). The relationship between the levels of PDGF-AB secretion and the levels of released phosphorylated-HSP27 was slightly weaken by the exclusion of non-responding samples, but was significant (R^2^ = 0.337, p = 0.001, n = 30) (Fig 4B).

### The relationship between individual parameters for platelet aggregation and the levels of released phosphorylated-HSP27 induced by collagen in type 2 DM patients

**Fig 5:**
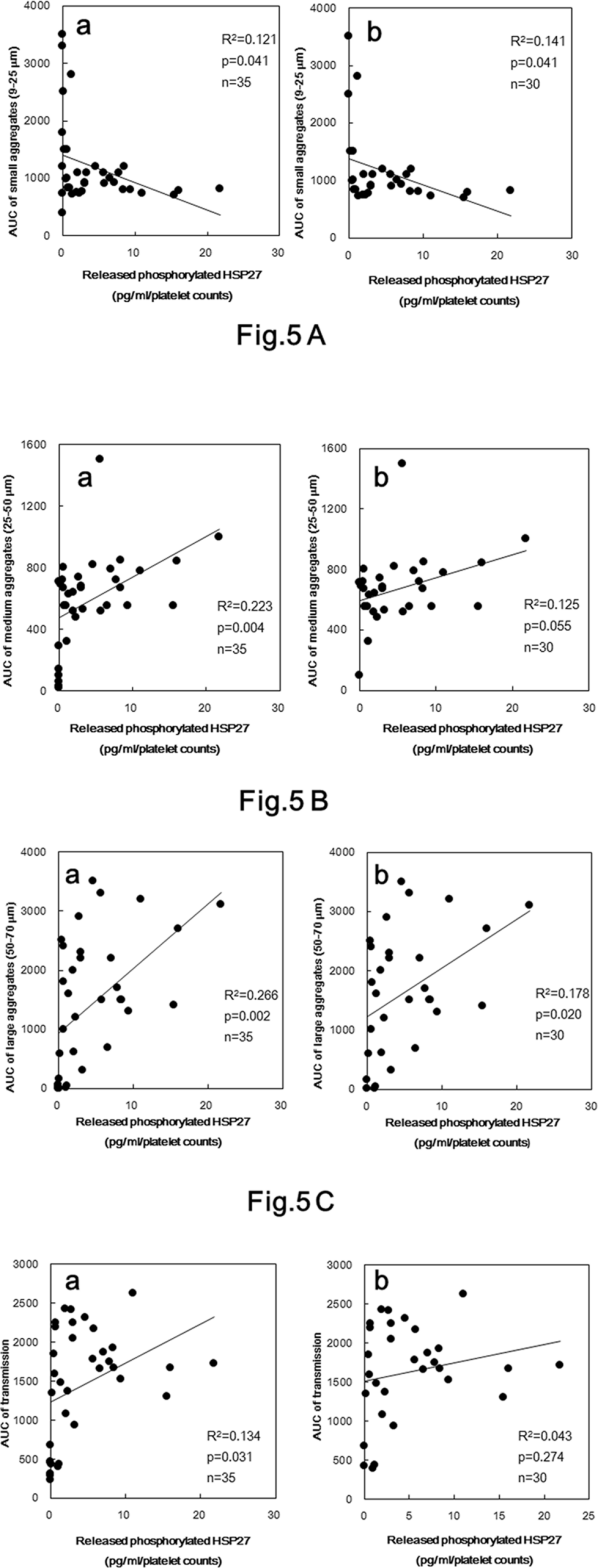
The relationship between individual levels of released phosphorylated-HSP27 and the area under the curve (AUC) of platelet aggregation induced by collagen in type 2 DM patients. The levels of released phosphorylated-HSP27 in the supernatant of the conditioned mixture after platelet aggregation stimulated by 0.3 μg of collagen for 30 min was determined using specific ELISA kits and data were collected with the platelet counts. AUC of platelet aggregation stimulated by 0.3 μg/ml of collagen for 4 min were determined by an aggregometer using LS system recorded individually by the size of aggregates, (A) AUC of small aggregates, (B) AUC of medium aggregates, (C) AUC of large aggregates, (D) AUC of total transmission. Each data were plotted and analyzed by linear regression analysis. (a) Whole subjects (n = 35) were plotted. (b) The residual subjects after excluding what concentration of phosphorylated-HSP27 could not be detected (n = 30) were plotted.

To investigate whether the platelet aggregation is related to the release of phosphorylated-HSP27 from platelets induced by collagen, we plotted the individual levels of area under the curve (AUC) of platelet aggregation classified by the size of aggregates against the levels of released phosphorylated-HSP27 induced by 0.3 μg/ml collagen in the platelets of type 2 DM patients. AUC of small aggregates (9–25 μm) induced by 0.3 μg/ml collagen was inversely proportional to the levels of extracellular phosphorylated-HSP27 (R^2^ = 0.121, p = 0.041, n = 35) (Fig 5Aa). On the other hand, AUC of medium aggregates (25–50 μm), large aggregates (50–70 μm) and light transmittance were directly proportional to the levels of released phosphorylated-HSP27 (R^2^ = 0.223, p = 0.004; R^2^ = 0.266, p = 0.002; R^2^ = 0.134, p = 0.031, respectively, n = 35) (Fig 5Ba, Fig 5Ca and Fig 5Da). The inversely proportional relationship between AUC of small aggregates and the levels of released phosphorylated-HSP27 was little affected even by the exclusion of non-responding samples (R^2^ = 0.141, p = 0.041, n = 30) (Fig 5Ab). The directly proportional relationship between AUC of large aggregates and the levels of released phosphorylated-HSP27 was slightly weaken by the exclusion (R^2^ = 0.178, p = 0.020, n = 30), but was significant (Fig 5Cb). The relationship between AUC of medium aggregates and the levels of released phosphorylated-HSP27 was weaken by the exclusion (R^2^ = 0.125, p = 0.055, n = 30) (Fig 5Bb). The relationship between light transmittance and the levels of released phosphorylated-HSP27 disappeared after the exclusion (R^2^ = 0.043, p = 0.274, n = 30) (Fig 5Db).

### The effect of exogenous recombinant phosphorylated-HSP27 on the platelet aggregation and the secretion of PDGF-AB induced by collagen

**Fig 6:**
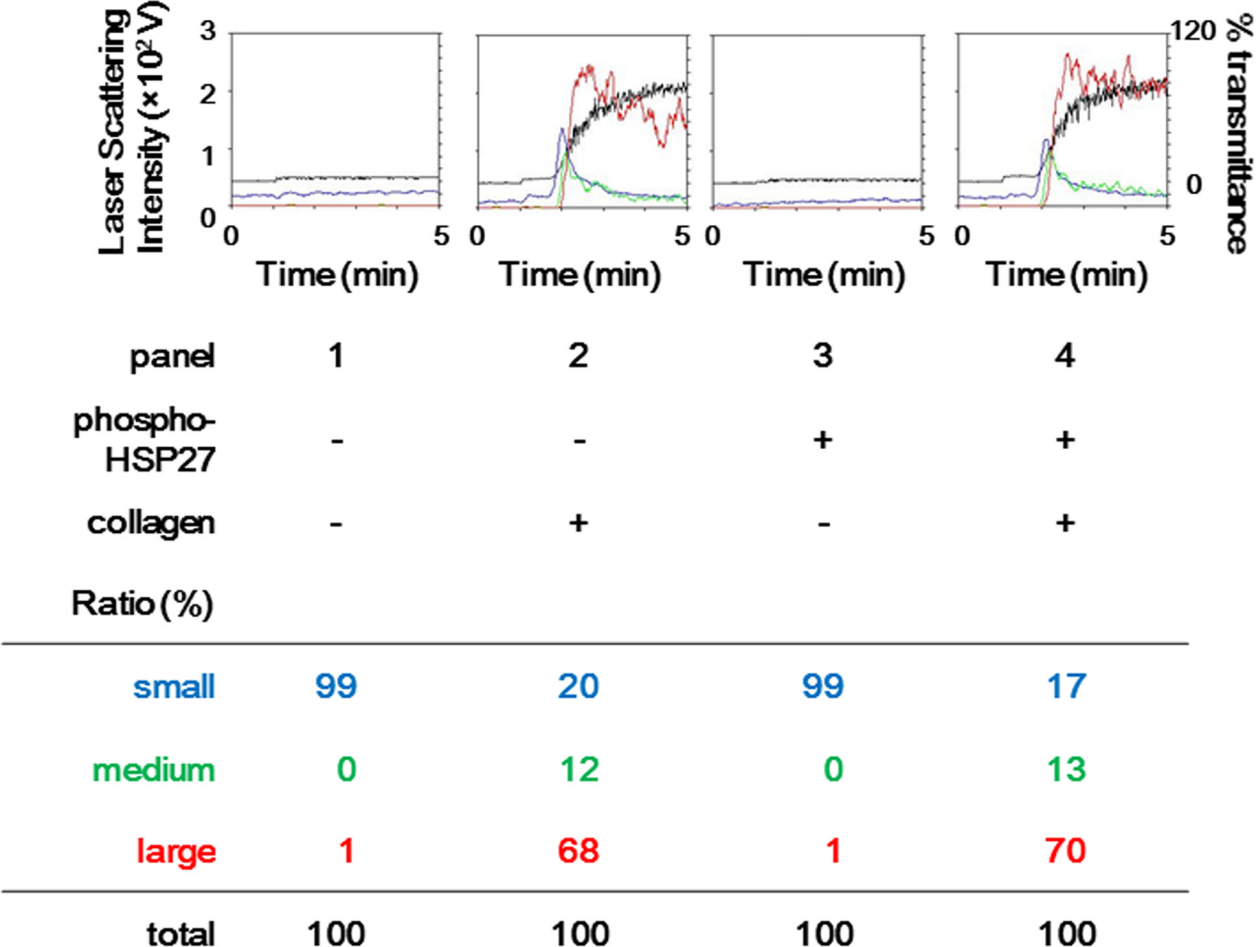
The effect of exogenous recombinant phosphorylated-HSP27 on the platelet aggregation induced by collagen in healthy subjects. Representative patterns of platelet aggregation as detected by an aggregometer with laser-scattering system are presented. PRP from healthy subjects was stimulated by 1 μg/ml of collagen or vehicle with 3 μg/ml of recombinant phosphorylated-HSP27 or vehicle in an aggregometer at 37°C for 4 min with a stirring speed of 800 rpm. The black line indicates the percentage of transmittance of each samples (the isolated platelets were recorded as 0%, and platelet free plasma was recorded as 100%). The blue line indicates small aggregates (9–25 μm); green line, medium aggregates (25–50 μm); red line, large aggregates (50–70 μm). The distribution (%) of aggregated particle size measured by laser-scattering method was presented as a lower panel.

We further examined the effects of exogenous recombinant phosphorylated-HSP27 on the aggregation and the release of PDGF-AB induced by collagen in the platelet derived from 3 healthy donors. Recombinant phosphorylated-HSP27 (3 μg/ml) had little effect on the collagen (1.0 μg/ml)-induced platelet aggregation (Fig 6). In the case, the ratio of large aggregates stimulated by 1 μg/ml of collagen alone was 68%, whereas the ratio stimulated by 1 μg/ml collagen and 3 μg/ml of recombinant phosphorylated-HSP27 was 70% according to the analysis of the size of the platelet aggregates. The exogenous recombinant phosphorylated-HSP27 neither affected the release of PDGF-AB with nor without collagen stimulation (control, 53.3 ± 62.9 pg/ml/platelet counts; 1.0 μg/ml of collagen alone, 243.6 ± 107.4 pg/ml/platelet counts; 3 μg/ml of phosphorylated-HSP27 alone, 32.7 ± 29.6 pg/ml/platelet counts; 1.0 μg/ml of collagen and 3 μg/ml of phosphorylated-HSP27, 279.3 ± 72.1 pg/ml/platelet counts, not statistically significant vs. the value of collagen alone).

## Discussion

In type 2 DM patients, we have previously reported that the collagen-induced activation of p44/p42 MAP kinase and p38 MAP kinase is implicated in the platelet hyper-aggregation [9]. The activation of p38 MAP kinase is reportedly regulates HSP27 phosphorylation in human platelets [10], [18], and we have recently reported that Rac regulates collagen-induced HSP27 phosphorylation via p44/p42 MAP kinase in human platelets, leading to the secretion of PDGF-AB [19]. On the basis of these findings, we investigated the relationship between platelet activation and HSP27 phosphorylation in type 2 DM patients. First, we showed that collagen truly induced the phosphorylation of HSP27 at Ser-78 and Ser-82 in a dose-dependent manner in the range between 0.1 and 1.0 μg/ml, and that the total HSP27 levels were reversely decreased in parallel with exhibiting large platelet aggregates (50–70 μm) induced by low dose of collagen (0.3 μg/ml). We confirmed that the levels of P2Y12 receptors did not exhibit significant change by the collagen stimulation in the platelets. We next clarified the relationship between the levels of phosphorylated-HSP27 (Ser-78) and the change of HSP27 protein levels individually in type 2 DM patients, and found that there was a significant inverse correlation in each other. These results lead us to speculate that HSP27 could be released into extracellular space from the collagen-stimulated platelets due to the phosphorylation.

Thus, we measured the released phosphorylated-HSP27 under the stimulation of low dose (0.3 μg/ml) collagen, and investigated the relationship between the individual levels of extracellular phosphorylated-HSP27 and intracellular collagen-induced phosphorylation of HSP27 in the platelets derived from type 2 DM patients. As a result, the levels of released phosphorylated-HSP27 stimulated by collagen was directly proportional to the levels of intracellular phosphorylated-HSP27, suggesting that the collagen-induced phosphorylation of HSP27 truly causes the release of HSP27 from the activated platelets into circulation. The association between intrinsic and released levels of phosphorylated-HSP27 is weak even though the deletion of non-responding (not releasing phosphorykated-HSP27) samples. It is possible that the phosphorylation of HSP27 is required but not sufficient for the release from the collagen-activated platelets from type 2 DM patients. In addition, it is known that HSP27 in plasma is predominantly existed as an unphosphorylated form, and that the levels are ng/ml order [23]. Thus, we could not specifically detect the release of unphosphorylated-HSP27 from platelets, even if the release was elicited by the activation of platelets. In healthy donors, we found that the levels of low dose collagen-stimulated release of HSP27 was detectable but relatively small in non-DM subjects compared with that in type 2 DM patients. Thus, it is likely that the collagen-stimulated release of phosphorylated-HSP27 associated with platelet aggregation could be characteristic in type 2 DM patients. We previously reported that collagen induces the activation of p38 MAP kinase and p44/p42 MAP kinase in the platelets derived from type 2 DM patients [9], and that collagen-induced activation of p44/p42 MAP kinase results in the phosphorylation of HSP27 in human platelets, resulting in the secretion of PDGF-AB [19], known as a mitogenic mediator promoting atherosclerosis [1]. We further investigated the relationship between the individual levels of collagen-induced release of phosphorylated-HSP27 and secretion of PDGF-AB from the platelets derived from type 2 DM patients, and found the close correlation in each other. Moreover, we showed that the levels of released phosphorylated-HSP27 induced by low-dose (0.3 μg/ml) collagen were significantly correlated with the AUC of platelet aggregation in type 2 DM patients. Interestingly, the relationships were different among the size of platelet aggregates. AUC of small aggregates (9–25 μm) was inversely proportional whereas that of large aggregates (50–70 μm) was directly proportional. Therefore, it is probable that the release of phosphorylated-HSP27 could reflect the accelerated aggregation of platelets in the pathological state of type 2 DM. Furthermore, we found that recombinant phosphorylated-HSP27 had little effect on the platelet aggregation or the secreted levels of PDGF-AB induced by collagen. In addition, we have previously reported that HSP27 hardly affected platelet aggregation by thrombin [24]. Based on our findings, it seems unlikely that the collagen-stimulated release of phosphorylated-HSP27 could affect platelet activation.

Accumulating evidence indicates that HSP27 not only intracellularly acts as a molecular chaperone but also extracellularly functions [10]. HSP27 released from macrophage reportedly has a protective effect against the development of atherosclerosis [25]. In addition, it has been reported that HSP27 upregulates not only anti-inflammatory factors such as interleukin (IL)-10 but also pro-inflammatory factors including IL-1β via the activation of NF-κB in macrophages [26]. Recently, it has been reported that human myocardium after global ischemia releases HSP27, and that HSP27 exhibits the pro-inflammatory effect through toll-like receptor (TLR)-2 and TLR4 in mouse coronary vascular endothelial cells [27]. On the other hand, it is well recognized that platelet activation originates from the initial tethering at the injured vascular sites exposing a subendothelial collagen [1], [2]. Based on our present findings, it is possible that the phosphorylated-HSP27, which is released from collagen-activated platelets accompanied with PDGF-AB might play a role in the development of atherosclerosis in type 2 DM patients. In addition, the phosphorylation of HSP27 in the platelets of these patients might be an important therapeutic target or a clinical indicator of the accelerated platelet aggregation, resulting in vascular disorders. Further investigation would be required to clarify the exact roles of released phosphorylated-HSP27 from human platelets.

In conclusion, our present findings strongly suggest that HSP27 is released from human platelets accompanied with its phosphorylation induced by collagen, which is correlated with the acceleration of platelet aggregation in type 2 DM patients.
